# The use of non-mydriatic ocular fundus imaging in the emergency department for detection of ocular syphilis presenting as presumed “papilledema”: a case series

**DOI:** 10.3389/fneur.2026.1881880

**Published:** 2026-07-30

**Authors:** Wei-Hsuan Lee, Mung Yan Lin, Etienne Bénard-Séguin, Andrew M. Pendley, Nancy J. Newman, Valérie Biousse

**Affiliations:** 1 Department of Ophthalmology, Emory University School of Medicine, Atlanta, GA, United States; 2 Department of Surgery, Section of Ophthalmology, University of Calgary, Calgary, AB, Canada; 3 Department of Emergency Medicine, Emory University School of Medicine, Atlanta, GA, United States; 4 Department of Neurology, Emory University School of Medicine, Atlanta, GA, United States; 5 Department of Neurological Surgery, Emory University School of Medicine, Atlanta, GA, United States

**Keywords:** disc edema, emergency department, maculopathy, non-mydriatic ocular fundus imaging, ocular syphilis, optical coherence tomography, papilledema

## Abstract

**Background:**

Ocular syphilis and neurosyphilis can present as seemingly “isolated” bilateral optic disc edema mimicking papilledema from intracranial hypertension. As a result, patients are often sent to the emergency department (ED) for “papilledema work-up,” which can lead to delayed or missed ocular syphilis diagnosis since serologic testing for syphilis is not routinely performed in patients with presumed “papilledema.” Our study describes eight patients initially referred to our ED for “papilledema” workup who were ultimately diagnosed with ocular syphilis based on characteristic findings seen on non-mydriatic ocular fundus photography and optical coherence tomography nerve/macula (NMFP-OCT) obtained in the ED.

**Objective:**

To describe the ocular imaging findings seen on non-mydriatic ocular fundus photography and optical coherence tomography (NMFP-OCT) in the ED and clinical outcomes of patients with ocular syphilis who presented to the ED with presumed “papilledema”.

**Materials and methods:**

Descriptive case series study involving prospective collection of all consecutive adult patients presenting to our general ED between 6/14/2023 to 9/21/2025 for “papilledema work-up,” who were subsequently diagnosed with ocular syphilis. Clinical findings on NMFP-OCT were reviewed.

**Results:**

From 6/14/2023 to 9/21/2025, 8/318 patients (2.5%) who underwent our “papilledema protocol” were ultimately diagnosed with ocular syphilis. All 8 patients had bilateral optic disc edema on ocular imaging, triggering our “papilledema protocol” in the ED. Visual acuities ranged from 20/20 to hand motion. 5/8 (63%) patients had outer retinal abnormalities and 7/8 (88%) had vitreous cells, which were remotely identified on NMFP-OCT imaging obtained in the ED by ED staff. 7/8 (88%) patients underwent attempted lumbar puncture (LP), but LP was unsuccessful in 1/7 patients. 5/6 (83%) patients with LP performed in the ED had normal CSF-opening pressure; 4/6 (67%) had elevated CSF nucleated cells [range 7–24]; 3/6 (50%) patients had reactive CSF VDRL. All patients had serologic positive RPR/syphilis IgG-IgM. All patients received 14 days of IV penicillin G. 4/8 (50%) patients were lost to follow-up. Among patients who were seen in outpatient follow-up, ranging from 2 weeks to 8 months after initial presentation, all were found to have improving or resolution of disc edema.

**Conclusion:**

Syphilis may present as seemingly “isolated” bilateral disc edema in the ED. Remote interpretation of NMFP-OCT obtained in the ED allowed for detection of subtle outer retinal changes and vitreous cells, heightening suspicion for ocular syphilis and triggering confirmatory serologic testing.

## Introduction

The burden of syphilitic infection in the United States is substantial, with 190,242 cases reported in 2024, which was a remarkable 42% increase in cases compared to 2020 (1). If not diagnosed and treated expeditiously, this disease, caused by *Treponema pallidum*, can be vision- and life- threatening (2). Notably, patients who present with ocular signs of syphilis might not present with concomitant systemic symptoms, which makes ocular syphilis a disease that requires a high index of suspicion to prompt further confirmatory serologic testing (3–6).

Ocular manifestations can present at any of the four stages of a syphilitic infection, making timely diagnosis extremely challenging (5, 6). While posterior uveitis and panuveitis are the most common presentations, syphilis can also manifest as anterior uveitis, chorioretinitis and retinal vasculitis, earning the disease entity its reputation as the “great mimicker” (7–12).

Importantly, syphilis can also manifest as a seemingly “isolated” bilateral anterior optic neuropathy (3, 13, 14), prompting referrals to emergency departments (ED) for presumed “papilledema” (i.e., bilateral disc edema initially felt to be consistent with papilledema in the setting of elevated intracranial pressure). This can result in delayed or even missed diagnoses as syphilis testing is not systematically performed as part of the standard “papilledema work-up.” Thus, early recognition of syphilis-related disc edema is crucial in timely treatment to prevent vision loss and other systemic comorbidities.

While ocular syphilis and neurosyphilis are technically distinct entities, they can occur concurrently, especially when there is posterior segment involvement such as in posterior uveitis or panuveitis (15), but there can also be overlap, such as in the setting of syphilis-associated optic neuropathy, which exists at the intersection of the two entities. Because it has been shown that cerebrospinal fluid (CSF) analysis may be abnormal in up to 70% of patients with ocular syphilis (16, 17), the general consensus is that ocular syphilis be treated as a neurosyphilis-equivalent entity with 10 to 14 days of intravenous (IV) penicillin therapy (18), even when CSF analysis is normal and CSF VDRL is negative (2, 19). For these reasons, as of 2021, the Center for Disease Control (CDC) no longer recommends obtaining lumbar puncture in patients presenting with presumed isolated ocular syphilis unless other neurologic abnormalities are present (20).

In June 2023, we instituted the routine use of a combined nonmydriatic fundus photography (NMFP) and optical coherence tomography (OCT) camera (NMFP-OCT) in our general university-based ED for patients presenting with visual and neurologic symptoms, including referrals for possible “papilledema” (21, 22). Remote interpretation of these ocular imaging studies is helpful in guiding initial workup in the ED prior to any in-person ophthalmologic consultation. We aimed to better delineate the ocular imaging features of patients presenting with presumed “isolated” bilateral optic disc edema who were subsequently diagnosed with ocular syphilis, with a focus on the diagnostic utility of nonmydriatic OCT in our quaternary care general ED. We hypothesized that NMFP-OCT imaging can be a valuable tool for the visualization of vitreous cells (indicative of intraocular inflammation) and of outer retinal changes suggestive of ocular syphilis in patients presenting with presumed “isolated” bilateral optic disc edema from ocular syphilis, particularly in the ED setting.

## Methods

In June 2023, the hybrid Topcon Maestro2 camera (Japan), which takes nonmydriatic true color 45-degree ocular fundus photographs with optical coherence tomography (OCT) of the optic nerve and macula, was installed in our general university-based ED (21, 22). In conjunction with the arrival of the camera, we developed an ED triage protocol in which the triage nurse systematically ordered NMFP-OCT for all patients presenting with visual and neurologic symptoms, including concerns for papilledema. Patients who were identified as having “papilledema-related” concerns underwent NMFP-OCT without pharmacologic dilation of the pupils performed by trained ED staff. By utilizing the Digital Imaging and Communications in Medicine (DICOM) standardized format, the images were automatically transferred from the camera to each patient’s electronic health medical record (EPIC), thereby made accessible to our institution’s providers for on-site or remote review. ED providers were asked to page the on-call ophthalmologist if they required an expedited real-time interpretation of the images.

Since the implementation of the camera in the ED, we prospectively collected all consecutive adult patients who presented to our academic medical center’s ED and underwent NMFP-OCT imaging from 6/14/2023 to 9/21/2025. Those patients who underwent our ED’s “papilledema protocol” were identified. Our “papilledema protocol” consists of blood pressure monitoring, MRI of the brain and orbits, without and with contrast, MRV of the head, lumbar puncture with opening pressure and cerebrospinal fluid evaluation, complete blood count and neurologic and neuroophthalmologic consultations.

Patients with a final diagnosis of ocular syphilis were further identified from this database. The diagnosis of ocular syphilis was predicated on a positive serum rapid plasma regain (RPR) and serum treponemal IgG/IgM, exclusion of alternative diagnoses, compatible ocular findings and imaging, consensus regarding diagnosis with the infectious disease service as well as response to penicillin treatment while inpatient. Lumbar puncture with CSF confirmation of neurosyphilis was not required for study inclusion, as the infectious disease service occasionally deferred lumbar puncture when it would not change patients’ management. HIV testing was systematically performed in all patients with suspected ocular syphilis. Information such as key neuroimaging and laboratory results, treatments, and outcomes were collected and analyzed. Ocular imaging studies were reviewed by two ophthalmologists to characterize and describe OCT findings, including findings related to the patient’s syphilis diagnosis. OCT findings of outer retinal abnormalities and posterior vitreous cells triggered serologic testing for syphilis, specifically RPR and syphilis IgG and IgM.

## Results

**Figure 1:**
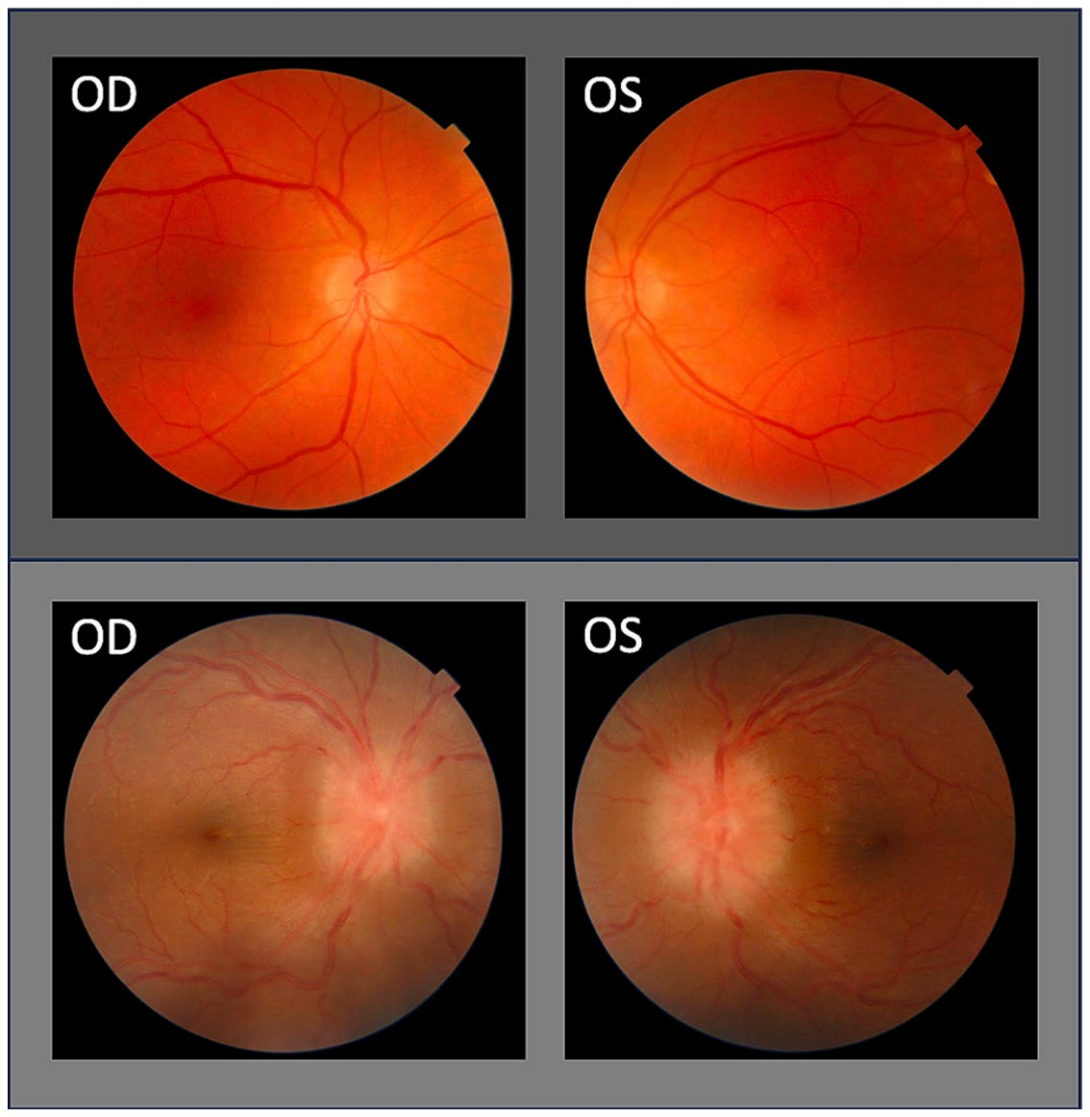
Non-mydriatic color fundus photographs demonstrating the range of bilateral disc edema severity in ocular syphilis patients presenting to our emergency department.

**Figure 2:**
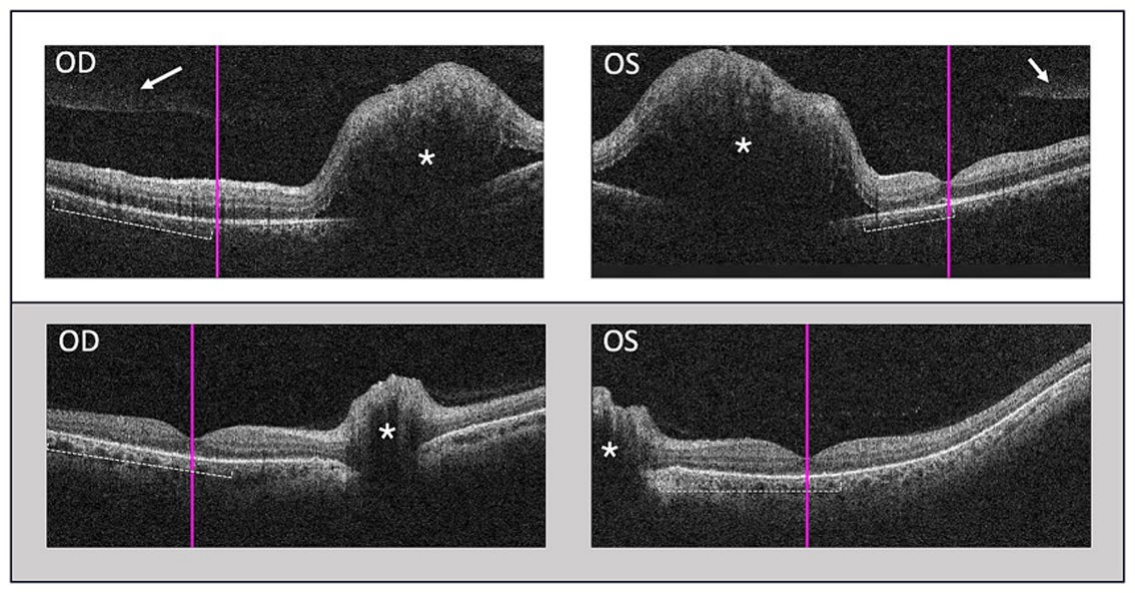
Optical coherence tomography (OCT) imaging of the optic nerves and macula revealing ocular inflammation seen as multiple hyperreflective foci within the posterior vitreous (white arrows) and outer retinopathy (dashed brackets) with focal disruption and irregularity of the retinal pigment epithelium. Note the swollen optic nerves in both eyes (asterisks). OD: right eye; OS: left eye.

From 6/14/2023 to 9/21/2025, 8/318 patients (2.5%) who underwent our papilledema protocol were ultimately diagnosed with ocular syphilis (Tables 1, 2). All had bilateral optic disc edema mimicking “papilledema” (Figure 1). Visual acuities ranged from 20/20 to hand motion. 5/8 (63%) patients had outer retinal abnormalities, specifically ellipsoid zone disruption and retinal pigment epithelial irregularity and 7/8 (88%) had posterior vitreous cells (Figure 2), all remotely identified by the on-call ophthalmologist on nonmydriatic NMFP-OCT imaging obtained in the ED by ED staff. All patients had normal brain imaging in the ED. 7/8 (88%) patients had a lumbar puncture (LP) attempted in the ED, but lumbar puncture was unsuccessful in 1/7 (88%) patients. CSF opening pressure was measured in 5/6 (83%) patients and found to be normal in all of them; 4/6 (67%) had elevated cells in the CSF [range 7–24]; 3/6 (50%) patients had reactive CSF-VDRL and were diagnosed with concurrent neurosyphilis. All patients were found to be serologic positive for serum RPR and syphilis IgG-IgM. All patients received 14 days of IV penicillin G. All patients who underwent a lumbar puncture were initiated on IV penicillin upon positive serologic syphilis testing prior to confirmation of CSF VDRL status. 2/8 (25%) of patients received a concomitant new diagnosis of HIV. 4/8 (50%) patients were lost to follow-up. Of the patients seen in follow-up, all had eventual resolution of disc edema and improvement of visual function. Of the four patients seen in follow-up, duration of follow-up ranged from 2 weeks to 8 months with demonstrated improvement or resolution of disc edema.

**Table 1:** Diagnostic clinical and laboratory characteristics of patients with ocular syphilis mimicking isolated papilledema.

Clinical or laboratory characteristic	Outcome
Visual acuity (eye with worse visual acuity)	20/20 to 20/90: 6/8 (75%) 20/90 to hand motion: 2/8 (25%)
HVF MD (OD/OS)	Median: −4.27 [−8.24,2.35]
Bilateral optic disc edema	Yes: 8/8 (100%)
Outer retinal abnormalities	Yes: 5/8 (63%) No: 3/8 (37%)
Presence of vitreous cells	Yes: 7/8 (88%) No: 1/8 (12%)
CSF opening pressure	Normal (<25 cm H_2_O): 5/5 (100%)
CSF nucleated cells	Elevated [Range 7–24] (>5):4/6 (67%) Normal (<5): 2/6 (33%)
CSF VDRL	Reactive: 3/6 (50%) Non-reactive: 3/6 (50%)
Serologic RPR/syphilis IgG, IgM	Positive: 8/8 (100%)
HIV status	Positive: 1/8 (12.5%) Negative: 7/8 (87.5%)
Treatment	14 days of IV-penicillin G: 8/8 (100%) Steroids (oral/drops): 3/8 (38%) Doxycycline: 3/8 (38%)
Outcome	Disc edema improved: 4/4 (100%) Loss to follow-up: 4/8 (50%)

Data are presented as *n* (%). HVF, Humphrey visual field (24–2 SITA-FAST program); CSF, cerebrospinal fluid.

**Table 2:** Case-by-case clinical characteristics and diagnostic findings.

Patient	Age	Sex	Symptoms	VA	Ocular findings	OCT findings	RPR titer	Trep Test	HIV	CSF OP (cm H_2_O)	CSF cell count	CSF VDRL
1	22	M	Headache and vision loss	OD 20/20 OS 20/20	Vitreous cells, disc edema	Vitreous cells and outer retinal changes	>1:256	Pos	Pos	17	7	Neg
2	57	M	Headache and vision loss	OD 20/30 OS 20/50	Vitreous cells, disc edema, placoid lesions	Outer retinal changes	1:128	Pos	Neg	16	1	Neg
3	60	M	Photopsia and floaters	OD 20/20 OS 20/15	Vitreous cells, disc edema, vasculitis	Vitreous cells	1:128	Pos	Neg	16	2	Pos
4	53	M	Vision loss	OD 20/100 OS HM	Vitreous cells, disc edema, placoid lesions	Vitreous cells, outer retinal changes	1:128	Pos	Neg	NA	NA	NA
5	55	F	Vision loss and photopsia	OD 20/30 OS 20/20	Vitreous cells, disc edema, macular edema	Vitreous cells, macular edema	1:128	Pos	Neg	18	7	Neg
6	35	F	Vision loss and floaters	OD 20/80 OS 20/70	Anterior chamber and vitreous cells, disc edema, retinal lesions	Vitreous cells, outer retinal changes	1:64	Pos	Neg	NA	NA	NA
7	51	M	Headache and vision loss	OD 20/100 OS 20/25	Anterior chamber and vitreous cells, disc edema	Vitreous cells, outer retinal changes	1:128	Pos	Neg	19	11	Pos
8	44	M	Vision loss, photophobia, eye pain	OD 20/20 OS 20/20	Anterior chamber and vitreous cells, disc edema	Vitreous cells	1:128	Pos	Neg	Not measured	23	Pos

## Discussion

We report eight patients referred to our ED for “papilledema” workup who were ultimately diagnosed with ocular syphilis due to suspicious characteristic findings seen on NMFP-OCT performed in the ED. Syphilis remains an important diagnostic consideration in neuro-ophthalmology given its ability to masquerade as presumed “isolated” bilateral disc edema (3, 13, 14). In such cases, patients often do not present with other systemic symptoms of syphilis, and neuroimaging and “standard” CSF analysis may be unrevealing. Thus, ancillary testing with OCT can be invaluable in revealing subtle outer retinopathy and inflammatory vitreous cells characteristic of ocular syphilis rather than isolated papilledema (23, 24), thereby triggering serum syphilis testing not routinely part of our “papilledema protocol”.

Importantly, even with in-person ophthalmological examination, these findings are often difficult to appreciate when using standard indirect or direct ophthalmoscopy alone, particularly in the ED. Furthermore, when a portable slit lamp is available for higher-magnification examination of the fundus, there can be significant inter-examiner variability in detecting the presence of inflammatory vitreous cells or retinal abnormalities, especially if findings are subtle. Therefore, nonmydriatic ocular imaging, particularly with OCT, in the ED setting can provide useful ancillary information, independent of in-person ophthalmological examination, for raising concern for syphilis and expediting its treatment.

There are some study limitations to consider. Given that this is a single-center quaternary academic ED, the prevalence of ocular syphilis presenting as seemingly “isolated” bilateral disc edema observed in our cohort in our ED may not be generalizable to other clinical settings. We did not systematically screen for syphilis in every patient who underwent the papilledema protocol because in our experience, patients almost always present with other ocular findings, which may be subtle but are present nonetheless on in-person examination and/or ancillary ocular imaging. Syphilis testing was only triggered at the discretion of the on-call ophthalmologist or neuro-ophthalmologist when findings suspicious for ocular syphilis were seen on remote interpretation of NMFP-OCT or in-person clinical examination at initial evaluation and during followup visits. Therefore, there was no comparative group to ascertain the prevalence of outer retinal changes or vitreous cells in patients presenting with “isolated” bilateral disc edema for non-syphilitic etiologies. Furthermore, our study did not include a control group from pre-NMFP-OCT implementation, so we did not perform a comparative analysis on whether the institution of NMFP-OCT has expedited time to diagnosis of ocular syphilis presenting as presumed “papilledema.” It is also important to note that 50% of patients were lost to follow-up and therefore, we are unable to determine long-term treatment response to IV penicillin in a fraction of our study sample.

Nevertheless, in light of the rising prevalence of ED and inpatient neuro-ophthalmology consultations (25) and the concomitant decline in ophthalmology coverage, particularly in community hospitals (26), our study shows that NMFP-OCT can potentially be helpful in reducing the number of hospital transfers, enable remote interpretation of ocular imaging and reduce burnout for both on-call ophthalmologists and neuro-ophthalmologists. The successful implementation of a NMFP-OCT camera at our institution has previously been described (22), and is contingent upon careful camera selection tailored to the specific ED setting, extensive training of ED staff to operate the camera autonomously and development of standardized protocols to facilitate work-flow (27).

## Conclusion

Syphilis remains a critical vision- and life-threatening condition that can present as presumably paucisymptomatic “isolated” bilateral disc edema. Such patients often undergo workup for papilledema in the ED, which can lead to superfluous and unnecessarily invasive or costly testing, which can delay diagnosis.

This study demonstrates that remote interpretation of NMFP-OCT obtained in the ED by ED staff could be a useful tool in the clinician’s armamentarium for detecting ocular syphilis. Specifically, the ability to identify subtle inflammatory vitreous cells and syphilitic outer retinopathy can help in differentiating syphilis from other causes of bilateral disc edema, initiate timely systemic treatment and, ultimately, minimize the risk of visual or neurological sequelae.

However, futures multi-center studies with larger cohort sizes, comparison groups and cost analyses would better elucidate the utility of NMFP-OCT in expediting patient care and minimizing unnecessary healthcare expenditures.

## Data Availability

The raw data supporting the conclusions of this article will be made available by the authors, without undue reservation.

## References

[1] Center for Disease Control and Prevention STI Statistics. Sexually transmitted infections surveillance. (2024) https://www.cdc.gov/sti-statistics/annual/index.html (Accessed April 26, 2026).

[2] Chevalier FJ Bacon O Johnson KA Cohen SE. Syphilis: a review. JAMA. (2025) 334:1927–40. doi: 10.1001/jama.2025.17362, 41100079

[3] Orman G Sungur G. Ophthalmologic assessment of patients with syphilitic optic neuropathy. Ocul Immunol Inflamm. (2023) 31:760–7. doi: 10.1080/09273948.2022.2046791, 35442838

[4] Furtado JM Simões M Vasconcelos-Santos D Oliver GF Tyagi M Nascimento H . Ocular syphilis. Surv Ophthalmol. (2022) 67:440–62. doi: 10.1016/j.survophthal.2021.06.003, 34147542

[5] Koundanya VV Tripathy K. Syphilis ocular manifestations. In: StatPearls. Treasure Island (FL): StatPearls publishing. (2026) (Accessed March 22, 2026). Available online at: http://www.ncbi.nlm.nih.gov/books/NBK558957/32644383

[6] Dutta Majumder P Chen EJ Shah J Ching Wen Ho D Biswas J See Yin L . Ocular syphilis: an update. Ocul Immunol Inflamm. (2019) 27:117–25. doi: 10.1080/09273948.2017.1371765, 29020491

[7] Ye Z Yang M Zou Y Zhang J Deng J Zong Y . Syphilis and the eye: clinical features, diagnostic challenges, and evolving therapeutic paradigms. Pathogens. (2025) 14:852. doi: 10.3390/pathogens14090852, 41011752 PMC12472546

[8] Cantisani C Rega F Ambrosio L Grieco T Kiss N Meznerics FA . Syphilis, the great imitator—clinical and dermoscopic features of a rare presentation of secondary syphilis. Int J Environ Res Public Health. (2023) 20:1339. doi: 10.3390/ijerph20021339, 36674095 PMC9859468

[9] Zhang T Zhu Y Xu G. Clinical features and treatments of syphilitic uveitis: a systematic review and meta-analysis. J Ophthalmol. (2017) 2017:1–15. doi: 10.1155/2017/6594849, 28751982 PMC5511639

[10] Shughoury A Carr EW Moorthy RS. Rhegmatogenous retinal detachment in syphilitic uveitis: a case series and comprehensive review of the literature. Ocul Immunol Inflamm. (2024) 32:1302–13. doi: 10.1080/09273948.2023.2238810, 37549228

[11] Paulraj S Ashok Kumar P Gambhir HS. Eyes as the window to syphilis: a rare case of ocular syphilis as the initial presentation of syphilis. Cureus. (2020) 12:e6998. doi: 10.7759/cureus.6998, 32206462 PMC7077063

[12] Amaral C Joy L Jimenez H Cruz-Inñigo YJ Ulloa-Padilla JP Oliver AL. Syphilitic outer retinopathy: a case report and review of the literature. J Vitreoretin Dis. (2022) 6:63–70. doi: 10.1177/24741264211018300, 37007722 PMC9976216

[13] Kayabaşı M Köksaldı S Saatci AO Bajin MS. Presentation of ocular syphilis with bilateral optic neuropathy. Neuro-Ophthalmol. (2023) 47:274–80. doi: 10.1080/01658107.2023.2222800, 38130808 PMC10732629

[14] Klein A Fischer N Goldstein M Shulman S Habot-Wilner Z. The great imitator on the rise: ocular and optic nerve manifestations in patients with newly diagnosed syphilis. Acta Ophthalmol. (2019) 97:641–7. doi: 10.1111/aos.13963, 30328249

[15] Reid GA Halmagyi GM Whyte C McCluskey PJ. Ocular vs neurosyphilis. Are they the same? A guide to investigation and management. Eye. (2024) 38:2337–49. doi: 10.1038/s41433-024-03150-w, 38914721 PMC11306553

[16] Lapere S Mustak H Steffen J. Clinical manifestations and cerebrospinal fluid status in ocular syphilis. Ocul Immunol Inflamm. (2019) 27:126–30. doi: 10.1080/09273948.2018.1521436, 30230943

[17] Vadboncoeur J Labbé AC Fortin C Serhir B Rabia Y Najem K . Ocular syphilis: case series (2000-2015) from 2 tertiary care centres in Montreal, Canada. Can J Ophthalmol. (2020) 55:30–7. doi: 10.1016/j.jcjo.2019.05.009, 31712031

[18] Durand ML Barshak MB Sobrin L. Eye infections. N Engl J Med. (2023) 389:2363–75. doi: 10.1056/NEJMra2216081, 38118024

[19] Centers for Disease Control and Prevention Syphilis: diagnostic considerations. (2024). Available online at: https://www.cdc.gov/std/treatment-guidelines/syphilis.htm (Accessed June 14, 2026).

[20] Workowski KA Bachmann LH Chan PA Johnston CM Muzny CA Park I . Sexually transmitted infections treatment guidelines, 2021. MMWR Recomm Rep. (2021) 70:1–187. doi: 10.15585/mmwr.rr7004a1 PMC8344968 34292926

[21] McHenry JG Lin MY Pendley AM Yan KY Alencastro Landim G Shanmugam N . Nonmydriatic ocular fundus imaging on consecutive patients seeking treatment at a general emergency department with vision symptoms. Ophthalmology. (2026) 133:178–86. doi: 10.1016/j.ophtha.2025.09.029, 41076033

[22] Shanmugam N Lin MY McHenry JG Yan KY Duffield S Pendley AM . Nonmydriatic ocular fundus imaging in a general emergency department: feasibility and novel considerations for systematic screening. Am J Ophthalmol. (2026) 281:126–37. doi: 10.1016/j.ajo.2025.09.016, 40962132

[23] Sverdlichenko I McDonald HM Xie JSC Margolin EA. Macular optical coherence tomography findings in patients with syphilitic optic neuropathy—a case series and systematic review. J Neuroophthalmol. (2025) 45:137–44. doi: 10.1097/WNO.0000000000002188, 39252151

[24] Vaze A De Angelis RE Simões M Arantes TE Moreto R Oliver GF . Optical coherence tomography findings in ocular syphilis involving the posterior segment of the eye. Ocul Immunol Inflamm. (2022) 30:1464–70. doi: 10.1080/09273948.2021.1892152, 33856284

[25] Lin MY Dattilo M Pendley AM Yan KY Keadey MT Wright DW . Neuro- ophthalmology consultations in an academic medical center with an emergency department equipped with nonmydriatic fundus imaging. Ophthalmology. (2026) 133:31–8. doi: 10.1016/j.ophtha.2025.08.013, 40849090

[26] Mott M Henderer JKD Mozoli RA Patavina CF Rapuano CJ Wiggins RE. Who's on call? Emergency care crisis looms. EyeNet Mag. (2019):27–9.

[27] Berman G Pendley AM Wright DW Silverman R Kelley C Duran MR . Breaking the barriers: methodology of implementation of a non-mydriatic ocular fundus camera in an emergency department. Surv Ophthalmol. (2025) 70:153–61. doi: 10.1016/j.survophthal.2024.09.012, 39357747

